# Quality of primary care for resettled refugees in the Netherlands with chronic mental and physical health problems: a cross-sectional analysis of medical records and interview data

**DOI:** 10.1186/1471-2296-15-160

**Published:** 2014-09-23

**Authors:** Marije A van Melle, Majda Lamkaddem, Martijn M Stuiver, Annette AM Gerritsen, Walter LJM Devillé, Marie-Louise Essink-Bot

**Affiliations:** Department of Public Health, Academic Medical Center, University of Amsterdam, Amsterdam, The Netherlands; Department of Clinical Epidemiology, Biostatistics and Bioinformatica, Academic Medical Centre, University of Amsterdam, Amsterdam, The Netherlands; Epi Result, Louis Trichardt, South Africa; NIVEL (Netherlands Institute for Health Services Research), Utrecht, The Netherlands; Faculty of Social and Behavioural Sciences, University of Amsterdam, Amsterdam, The Netherlands; National Knowledge and Advisory Center on Migrants, Refugees and Health (Pharos), Utrecht, The Netherlands

**Keywords:** Chronic disease, Mental health, Refugees, Quality of care, Primary care

## Abstract

**Background:**

A high prevalence of mental and physical ill health among refugees resettled in the Netherlands has been reported. With this study we aim to assess the quality of primary healthcare for resettled refugees in the Netherlands with chronic mental and non-communicable health problems, we examined: a) general practitioners’ (GP) recognition of common mental disorders (CMD) (depression and anxiety, and post-traumatic stress disorder (PTSD) symptoms); b) patients’ awareness of diabetes type II (DMII) and hypertension (HT); and c) GPs’ adherence to guidelines for CMD, DMII and HT.

**Methods:**

From 172 refugees resettled in the Netherlands, interview data (2010–2011) and medical records (n = 106), were examined. Inclusion was based on medical record diagnoses for DMII and HT, and on questionnaire-based CMD measures (Hopkins Symptom Checklist for depression and anxiety; Harvard Trauma Questionnaire for PTSD). GP recognition of CMD was calculated as the number of CMD cases registered in the medical record compared with those found in interviews. Patient awareness of HT and DMII was scored as the percentage of subjects diagnosed by the GP who reported their condition during the interview. GPs’ adherence to guidelines for CMD, DMII and HT was measured using established indicators.

**Results:**

We identified 37 resettled refugees with CMD of which 18 (49%) had been recognised by the GP. We identified 16 refugees with DMII and 14 with HT from the medical record; 24 (80%) were aware of their condition. Thirty-five out of these 53 (66%) resettled refugees with chronic mental and non-communicable disorders received guideline-adherent treatment.

**Conclusion:**

This study shows that awareness in resettled refugees of GP diagnosed DMII and HT is high, whereas GP recognition of CMD and overall guideline adherence are moderate.

## Background

According to the United Nations Refugee Agency there are approximately 75,000 refugees (defined as “*persons granted a complementary form of protection and those granted temporary protection*”) living in the Netherlands [1]. When granted a residence permit, asylum seekers become permit holders and full resettlement is made possible. Then, resettled refugees are entitled to the same health care as any other Dutch citizen. Prior to becoming a permit holder, asylum seekers reside mainly in reception centres located throughout the Netherlands. Access to health care is broad but regulated in a different way: i.e. the first contact point with health services is the telephone line of the Asylum seekers’ Healthcare Centre (GCA), instead of the general practitioner (GP). The GCA is in charge of directing the health matter to a GP or another primary healthcare provider. With a (temporary or definitive) residence permit, resettled refugees (or permit holders) can make direct contact with their GP (as do the general Dutch population). In this transition information from the medical file is transferred to the GP. Generally, the GP plays a central role as a first contact point in the organisation and access to healthcare services in the Netherlands. Access to most specialist health services can be gained through referral from the GP.

In 2003–2004, the first wave of this study (T1) on health and healthcare utilisation of asylum seekers and permit holders in the Netherlands was conducted among 410 respondents from Afghanistan, Iran and Somalia [2]. The second wave (T2) evolved subsequently and was conducted in 2010–2011 among 172 of those latter respondents, all of whom had meanwhile obtained a permit. The data collection used in the second wave was an exact copy of the first.

Results of the first assessment (T1) showed a high prevalence of psychiatric disorders, including depression (68.1%), general anxiety (39.4%) and post-traumatic stress disorder (PTSD; 28.1%). These numbers far exceeded worldwide prevalence data (depression 5% [3], anxiety 10% and PTSD 6.8% [4]), which is comparable to the current prevalence in the Dutch population (depression: 6.1% (5), anxiety 10.1%, and PTSD 1.3%) [5]. This earlier study confirmed results from international studies on refugees in Western countries [6–9].

Guideline adherence for CMD the Netherlands is 27–58% [10], however previous research showed that refugees with a mental disorder are less likely to receive adequate care and/or a referral to mental health care services than the general population, even when controlled for socioeconomic factors [11].

Chronic non-communicable conditions are prevalent among refugees resettled in Western countries [12–14]. Depending on the populations and definitions, prevalence as high as 15.5% has been reported for diabetes type II (DMII) and 42% for hypertension (HT) [15], whereas in the native Dutch population this is 4.1% and 51%, respectively [16]. Among Dutch populations of African descent prevalences of DMII and HT are 46% and 38% respectively [17]. Guideline adherence for DMII and HT in the Netherlands is 41–59% and 60% respectively [10]; however previous studies found low diabetes control and low hypertension control in migrant populations, suggesting inadequate quality of care in these groups [18, 19]. In addition, one study found low control rates for diabetes among refugee populations, suggesting inadequate quality of care for members of this group [19]. However, this topic has not yet been thoroughly investigated.

Based on the relatively high disease burden of resettled refugees in the Netherlands, and the sparse evidence on primary healthcare for resettled refugees, this study aims to examine the quality of GP care for resettled refugees in the Netherlands. We focused on patients with DMII, HT and symptoms of a common mental disorder (CMD) because of the relatively high prevalence of these health problems in our research sample. Only data from the second wave of the study (T2) were used. We examined both interview data and GP medical records. Quality of care can be assessed in several ways. We chose to limit the assessment of quality of care to the performance of the practitioner [20] through guideline adherence. Rather than limiting the present study to the investigation of guideline adherence by GPs, we also addressed the extent to which the GP recognised or diagnosed the three health complaints (GP-recognition) that are the focus of this study and the extent to which resettled refugees were aware of their own chronic disease (patient awareness).

## Methods

### General study design

This study is a cross-sectional analysis of healthcare medical records and interview data, embedded in a prospective two-wave longitudinal cohort study on health and healthcare use by refugees in the Netherlands.

### Setting and study population

**Figure 1:**
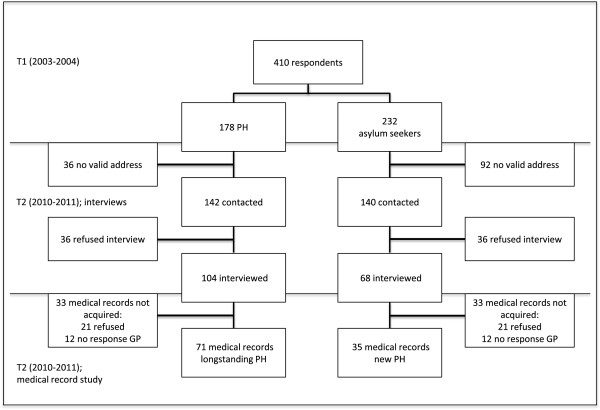
**Flowchart of respondents throughout the two waves of the study.** T1 had a total of N = 410 respondents, at T2 (the time of this study) 172 respondents participated in the interviews. 106 medical records were collected. PH = residence permit holders.

All 410 respondents of the first wave of the study (T1; 2003–2004) (for details on recruitment and methods see [21, 22]) were considered eligible for the second wave of this study (T2). Figure 1 presents an overview of the data collection process. Of those 410 original respondents, 7 years later, 282 had a valid address in the Netherlands. Of those 282 respondents, 172 participated in the second wave of the study (response = 61%, retention rate = 42%). All respondents in the second wave had meanwhile become permit holders (Figure 1).

The 172 participants at T2 were also asked for their permission to collect data from their GP medical records on the year preceding the interview date. 130 participants signed the informed consent (130/172, 81%). Finally, 106 (106/130, 82%) complete medical records were made available from several GP practices.

### Data collection

**Figure 2:**
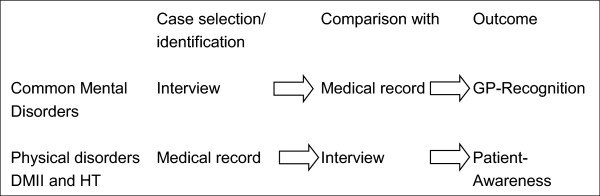
**Patient identification and measurement of cases.** For Common Mental Disorders, cases are selected in the interview and compared to the medical record (GP-recognition). Diabetes type II (DMII) and Hypertension (HT) cases are selected in the medical record and compared to the interview (patient-awareness).

This study focused on CMD, DMII and HT because of the widely accepted quality indicators for treatment and because of the high prevalence of these conditions among the present study population. Figure 2 shows the process of patient identification, data extraction and analysis for CMD, DMII and HT.

#### Patient interviews

Only interviews conducted at T2 were used for the present study. All interviews were questionnaire based and conducted in the language of choice of the participants (Dutch, Dari, Pashto, Farsi or Somali). Interviewers were matched on gender and ethnic background. The questionnaire was pre-tested in a panel with key-figures from several ethnical groups, and a random sample of respondents in this study. In the interview respondents were questioned about their health and healthcare utilisation. This included a list of common conditions in medical and common language.

#### Socio-demographic variables

Socio-demographic variables included in the presented analyses are: age; gender; country of birth; and educational level (‘none’; ‘religious school or primary school’; ‘secondary school’; ‘higher education’; and ‘vocational training and university’). Length of time in the Netherlands since receiving the permit was also recorded.

### GP’s medical records

- *Recognition of common mental disorders* The presence of a CMD was established during the interview using the Hopkins Symptom Check List for depression and anxiety (HSCL-25) and the Harvard Trauma Questionnaire for PTSD (HTQ); both questionnaires are validated for use in family practice and within several refugee groups [23–26]. However, the validity and reliability of the translations of these instruments (Dari, Pashto, Farsi and Somali) has not been tested in the population included in this study. We applied widely used cut-off points of 1.75 for the HSCL-25 or 2.5 for the HTQ.
- *Chronic non*-*communicable diseases* We reviewed medical records to identify patients with DMII and HT. A respondent was considered to have DMII when DMII was found in the problem list and/or the consultations, and/or when an anti-diabetic agent was prescribed.

### Quality of healthcare

- *GP recognition of CMD* GP recognition was calculated as the number of CMD cases registered in the medical record compared to those found in the interviews.
- *Patient awareness of DMII and HT* Patient awareness of DMII and HT was scored as the percentage of subjects with DMII or HT diagnosed by the GP who reported their conditions in the interview. Participants were explicitly asked to indicate if they had a chronic condition using a list of chronic conditions which included DMII and HT. Patient awareness of DMII and HT were recorded separately.
- *GPs’ guideline adherence* To evaluate GPs’ guideline adherence, we used existing quality indicators based on the guidelines of the Dutch College of General Practitioners [27]. Table  1 shows a summary of these indicators.

**Table 1:** **Indicators for guideline adherence used in our study**

**CMD:**	1) At least five additional GP consultations within the same illness episode
	2) And/or short/long-term prescription of antidepressants
	3) And/or a referral to a mental healthcare specialist.
**DMII:**	Compliance with at least four of the following eight rules, including testing in the past year
	1) HbA1c
	2) Serum LDL cholesterol
	3) Plasma creatinine level
	4) Proteinuria
	5) Blood pressure measurement
	6) Weight
	7) A foot examination
	8) Registration of a smoking habit.
**HT:**	Compliance with at least three of the following five rules based on the GP’s attention paid in the past year to
	1) (Compliance with) therapy
	2) Smoking behaviour (in case of current smoking or history of smoking)
	3) Blood pressure measurement
	4) Weight
	5) Lifestyle or modification thereof

- Assessment of guideline adherence in CMD used a set of indicators used in previous studies on guideline adherence in treatment of CMD [28–30] and was defined as: 1) at least five additional GP consultations within the same illness episode, 2) and/or short/long-term prescription of antidepressants, 3) and/or a referral to a mental healthcare specialist.
- Guideline adherence in DMII care was defined as compliance with at least four of the following eight rules [31], including testing in the past year of: 1) HbA1c; 2) serum LDL cholesterol; 3) plasma creatinine level; 4) proteinuria; 5) blood pressure measurement; 6) weight; 7) a foot examination; and 8) registration of a smoking habit. We used a strict scoring system of quality indicators (e.g. when “lab done” was noted in the record without specification, the quality indicators HbA1c, serum LDL cholesterol and plasma creatinine were scored as negative). To avoid an underestimation of guideline adherence, we chose a lenient cut-off value of 4 out of 8 criteria.
- Guideline adherence in HT care was defined as compliance with at least three of the following five rules [32] based on the GP’s attention paid in the past year to: 1) (compliance with) therapy; 2) smoking behaviour (in case of current smoking or history of smoking); 3) blood pressure measurement; 4) weight; and 5) lifestyle or modification thereof (e.g. physical activity advice, low salt intake, alcohol reduction).

### Statistical analysis

Descriptive statistics were used to present characteristics of the study groups, compared to characteristics of non-responders, and to evaluate GP recognition of CMD, patient awareness of chronic health problems (DMII and HT) and guideline adherence in all disorders. Group differences were tested using Fishers’ exact test for categorical data and an independent t-test for continuous data. All statistical tests were performed as two-tailed and p < 0.05 was considered statistically significant.

Statistical analyses were performed using SPSS 19.0 for Windows.

### Ethics

Our project was sponsored by the GGD Nederland (Association of Community Health Services in the Netherlands). According to Dutch law, this study was exempt of formal medical-ethical approval but because of the vulnerable legal position of asylum seekers [and refugees] formal approval was obtained before the first wave. Before the interview at T2 informed consent was obtained from all respondents.

## Results

Table 2 shows the characteristics of these 172 refugees, divided into groups for which we did and did not acquire a medical record. There were no significant differences between these groups, except that Somali refugees were underrepresented in the group with a medical record, i.e. 35% of the Somali patients did not give permission to collect their medical records, compared to 18% of the Afghan and 13% of the Iranian participants.

**Table 2:** **Characteristics of the participants (n = 172) with (n = 106) and without (n = 66) an available medical record**

	Medical record (n = 106)^1^	No medical record (n = 66)^1^	p-value
Age in years: mean (SD)	46.4 (12)	44.4 (13)	0.345
Gender (female): n (%)	51 (48%)	37 (56%)	0.311
Time since permit in years: median (range)	10 (1–26)	8.5 (1–23)	0.822
Country of origin: n (%)			
- Afghanistan	50 (47%)	32 (49%)	
- Iran	45 (43%)	18 (27%)	
- Somalia	11 (10%)	16 (24%)	0.023
Education: n (%)	(n = 105)	(n = 64)	
- Low	19 (18%)	13 (20%)	
- Intermediate	26 (25%)	14 (22%)	
- High	60 (57%)	37 (58%)	0.884
Prevalence			
- PTSD (HTQ score > 2.5) (n,%)	17 (16%)	9 (14%)	0.669
- Anxiety and/or Depression (HSCL-25 > 1.75)	37 (35%)	23 (35%)	0.994
- CMD (PTSD, depression or anxiety)	37 (35%)	23 (35%)	0.994
- Diabetes type II (according to interview)	14 (13%)	15 (23%)	0.352
- Hypertension (according to interview)	16 (15%)	15 (23%)	0.188

PTSD = Post-Traumatic Stress Disorder, HTQ = Harvard Trauma Questionnaire, HSCL = Hopkins Symptom Checklist, CMD = Common Mental Disorder.

^1^Proportion’s denominator, unless indicated otherwise.

Of all the 172 refugees participating in the interviews, the prevalence of CMD (anxiety, depression and PTSD), DMII and HT identified in the interview was 34.9% (60/172), 17% (29/172) and 18% (31/172), respectively.

### GP recognition of CMD

Of the 106 respondents for whom we had medical records, 37 (35%) had symptoms of CMD during the interview. Of these 37 respondents, the GP recognized a CMD in 18 (49%). Of the remaining 69 respondents, a further 6 (9%) were identified as having a CMD in the medical record, although they were not identified as having a CMD diagnosis in the interview (Table 3).

**Table 3:** **GPs’ recognition of chronic mental disorders (CMD)** ^**1**^ **; number of cases of CMD identified in the interview and as registered by the GP in the medical record**

	CMD diagnosis in medical record	No diagnosis of CMD in medical record	Total patients
CMD measured in interview	18 (49%)	19 (51%)	37
No CMD measured in interview	6 (9%)	63 (91%)	69
Total	24	72	106

^1^PTSD, depression or anxiety.

### Patient awareness of DMII or HT

Of the 106 respondents with an available medical record, 30 patients were documented to by the GP as having either DMII or HT. Of these 30 patients, 24 (80%) indicated awareness of their condition (Table 4). Of the 16 patients documented by the GP as having DMII, 15 (94%) indicated awareness of their condition. All refugees reporting DMII in the interview had a DMII diagnosis listed in their medical record. Of the 14 patients documented by the GP as having HT, 9 (64%) indicated awareness of their condition. Ninety-two respondents were not documented by the GP as having HT. Of these, 7 (8%) respondents reported to have HT during the interview.

**Table 4:** **Patient awareness of diabetes type 2 (DMII) and hypertension (HT) among resettled refugees (n = 106)**

	Diagnosis registered in medical record	No diagnosis registered in medical record	Total patients
DMII reported in interview	15 (94%)	0 (0%)	15
DMII not reported in interview	1 (6%)	90 (100%)	91
Total	16	90	106
HT reported in interview	9 (64%)	7 (8%)	16
HT not reported in interview	5 (36%)	85 (92%)	90
Total	14	92	106

Number of self-reported cases from the interviews and diagnoses as registered by the GP.

### Guideline adherence

Of the 54 refugees with a GP-diagnosed chronic disorder listed in the medical record, 35 (65%) were treated according to healthcare guidelines. Of the 24 refugees with a CMD diagnosed by the GP, 17 (71%) received guideline recommended care. Of the 30 refugees with DMII or HT, 18 (60%) received guideline recommended care: 10/16 (63%) for DMII and 8/14 (57%) for HT, respectively.

## Discussion

### Main findings

In this study, the GP recognition rate for CMD was 49%. Patient awareness for the chronic non-communicable diseases was 80% (94% for DMII and 64% for HT). The GPs’ adherence to guidelines for CMD, DMII and HT was 65%. All these percentages are similar to those found in the general Dutch population [10, 17, 29, 33–35].

### Strengths and limitations

A strength of this study is the combination of interviews and medical records as data source. These two sources together provide a more comprehensive view on the process of healthcare provision by the GP, from GP recognition to patient awareness, to guideline adherence. To our knowledge, this is the first study focusing on healthcare for refugees living in a Western country for a longer period of time. Also, our analyses were condition-specific rather than analysing general GP care, which allows a more focussed quality assessment of GP care.

This study also has some limitations. First, the results may be hampered by the small sample size, which often occurs in research on refugees. The prevalence of HT in the respondent group was lower than expected based on literature; this might be because healthy participants were more inclined to give permission to collect their medical records.

Also, in the present study, the refugees had lived in the Netherlands for a considerable period of time, had good command of the Dutch language, and had a relatively high education level. Patients with higher health skills may be more inclined to participate in research [36, 37], which may explain these high percentages of patient awareness. These factors might limit the generalisability of these results to other, or more recent, groups of refugees. However, CMD-rates in this subgroup of 172 respondents (47% in 2003) were comparable to the overall rates found in the first wave of the study (48%; N = 410), so the respondents in the second wave do not seem to differ from the respondents in the first wave [38].

In addition, the translations of the survey tools we used in the diagnoses of CMD were not validated which may have implications for the diagnosis of CMD in this study. This could have possibly resulted in false positive and/or false negative case findings.

Finally, the set of adherence indicators we used for CMD was rather loose compared to the adherence indicators for DMII and HT; they make no distinction between treatments. Every treatment (therapy or antidepressants) or more than five GP observations is seen as guideline adherence. This ignores the (over) prescription of antidepressants where psychotherapy would be optimal. Also, it is possible that a patient had over five appointments with the GP several in which a patient is returning because they do not feel their needs have been met, rather than pro-active follow-up or intervention by the GP.

### GP recognition rate of common mental disorders

In previous research, the GP recognition rates for CMD in the general Dutch population are below 50% depending on the definition used [29, 33, 35]. Our study shows a similar recognition rate of 49%.

### Patient awareness of chronic diseases

In the present study, the 80% patient awareness rate for DMII and HT was much higher than reported in other studies, ranging from 29–60% among Dutch patients [34], whereas the reported awareness of HT (64%) was similar to that in other ethnic populations living in the Netherlands [17]. No other studies were found reporting on awareness among patients with DMII. While awaiting confirmation in future studies, these figures are reassuring.

### Guideline adherence by GPs

In this study, the GP guideline adherence for refugee patients was found to be consistent with other reports of guidelines adherence in the Netherlands. A systematic review on guideline adherence in the Netherlands reported guideline concordant care for 27–58% of patients with a CMD, 41–59% for those with DMII, and 60% in the HT group [10]. This latter study suggests that no *specific* barriers exist in the use of GP services for refugees with chronic conditions compared to the Dutch general population.

### International/European comparison

Unfortunately, we found no international studies on the quality of primary care for resettled refugees in Western countries. To our knowledge, ours is the first study focusing on healthcare for refugees living in a Western country for a longer period of time. International studies on quality of care in minorities and refugees show that these groups are less likely to receive adequate care [11, 18, 19]. We can only speculate why our study shows a comparable quality of care to the native Dutch population in our population of resettled refugees. Our refugee population had lived in the Netherlands for a considerable period of time, had good command of the Dutch language, and had a relatively high education level. Patients with higher health skills in general are more inclined to participate in research. This can create a selection bias and explain our results. Another possibility is that barriers experienced by more recently arrived refugees (e.g. affordability, poor health literacy and understanding of the health system, medical mistrust, discrimination, and linguistic and cultural factors) [39] reduce through time. Refugees learn the language, culture and get to know the Dutch health care system. They build a relationship with their GP, which lowers mistrust.

Our results are of interest for all countries with a primary care-based healthcare system and we recommend further research on this resettled population, especially in view of the constant flow of refugees over time.

### Implications

The results of this study indicate that GP recognition of CMD, patient awareness of chronic non-communicable diseases, and guideline concordant care by the GP for resettled refugees with chronic health problems, is neither better nor worse than the care for ethnic Dutch patients. Nevertheless, GP awareness of the high prevalence of CMD (and especially PTSD) in this population could be further improved, together with overall patient awareness of HT.

Future research should combine assessment of primary healthcare provision for (resettled) refugees, native Dutch and other Dutch ethnicities in a matched controlled study to further explore known barriers and interventions needed.

## Conclusion

Our study shows that awareness among resettled refugees of GP diagnosed DMII and HT is high, whereas GP recognition of CMD and overall guideline adherence are moderate.

Recognition, awareness and guideline adherence were in concordance with that of the general Dutch population. However, GP recognition and patient awareness of CMD and HT are not yet optimal in either the refugee or the general patient population.
